# Reliability and acceptability of a five-station multiple mini-interview model for residency program recruitment

**DOI:** 10.3402/jchimp.v3i3-4.21362

**Published:** 2013-12-17

**Authors:** Julian Diaz Fraga, Adetokunbo Oluwasanjo, Thomas Wasser, Anthony Donato, Richard Alweis

**Affiliations:** 1 Department of Medicine, Jefferson Medical Center, Philadelphia, PA, USA; 2 Department of Medicine, Reading Health System, West Reading, PA, USA; 3 Consult-Stat, Macungie, PA, USA

**Keywords:** education, interviews, medical graduate, residency recruitment, reliability

## Abstract

**Background:**

Standard interviews are used by most residency programs in the United States for assessment of aptitude of the non-cognitive competencies, but variability of interviewer skill, interviewer bias, interviewer leniency or stringency, and context specificity limit reliability.

**Aim:**

To investigate reliability and acceptability of five-station multiple mini-interview (MMI) model for resident selection into an internal medicine residency program in the United States.

**Setting:**

One independent academic medical center.

**Participants:**

Two hundred and thirty-seven applicants and 17 faculty interviewers.

**Program description:**

Five, 10-min MMI stations with five different interviewers blinded to the candidate's records and one traditional 20-min interview with the program director. Candidates were rated on two items: interpersonal and communication skills, and overall performance.

**Program evaluation:**

Generalizability data showed that the reliability of our process was high (>0.9). The results of anonymous surveys demonstrated that both applicants and interviewers consider the MMI as a fair and more effective tool to evaluate non-cognitive traits, and prefer the MMI to standard interviews.

**Discussion:**

The MMI process for residency interviews can generate reliable interview results using only five stations, and it is acceptable and preferred over standard interview modalities by the applicants and faculty members of one US residency program.

In 1999, the Accreditation Council for Medical Education (ACGME) endorsed core competencies in six different areas to evaluate residents during training: patient care, medical knowledge, system-based practice, practice-based learning and improving, professionalism, and interpersonal and communication skills. Success in training is defined by achieving competence in the 6 core areas to the level expected of a new practitioner (1). Standard interviews are used by most residency programs in the United States for assessment of aptitude of the non-cognitive competencies such as professionalism, and interpersonal and communication skills. However, variability of interviewer skill, interviewer bias, interviewer leniency or stringency, and context specificity makes reliability too low for the ‘high-stakes’ resident selection process (2–10) leading one author to describe the process as an ‘elaborate, labor-intensive lottery’ (11).

The multiple mini-interview (MMI) model was first developed in 2001 by Eva et al., to mitigate interviewer bias and context specificity by increasing the number of interviewers and standardizing interview questions (12). Since then, the MMI model has been used as a recruitment tool in several medical schools and some residency programs in Canada and the United Kingdom (10, 13, 14). Evidence for its high reliability has been demonstrated using 6–12 interview stations (12, 15–17). Interviewers and applicants have found it to be an acceptable alternative to a traditional interview (13, 14, 18). The MMI has also shown predictive validity to clinical performance measures and licensing examination scores (19, 20).

The objective of this research was to investigate the acceptability and reliability of a five-station MMI model in selecting residents into an internal medicine residency program in the United States.

## Methods

This study was a non-randomized, retrospective cohort study analyzing the ratings and post-interview surveys from the applicants and interviewers of the residency interviews in a single, independent academic medical center.

In May of 2011, our resident selection committee proposed implementation of the MMI model to address our challenges in recruiting residents with the non-cognitive skills that we believed were critical to physicianship. We developed different scenarios that allowed for assessment of professionalism, communication skills, critical thinking, ethical behavior, tolerance for uncertainty, and teamwork. A traditional 20-min interview with the program director was maintained, though given less value in the creation of the rank order list, to assuage faculty concerns about the loss of some of the program-specific information that could be exchanged in that setting.

All interviewers received 2 hours of training in MMI concepts and logistics, which included practice during a simulated MMI station. The interviewer panel included faculty members and senior internal medicine resident volunteers.

On the interview day, each applicant completed five 10-min MMI stations and one traditional 20-min interview with the program director. MMI interviewers were blinded to the content of applicant files to minimize biases incurred by advance knowledge of the applicant. At each MMI station, applicants had 2 min to read background information on the scenario and 8 min to address the scenario with the interviewer. Interviewers spent a total of 90 min on each interview session. Separate rooms and a single interviewer were assigned to each station. At the end of each station, candidates were rated on two items, interpersonal and communication skills, and overall performance, using a seven-point anchored Likert scale. Interviewers were also allowed to note any ‘red flag’ issues in a free-text commentary field.

Applicants were surveyed anonymously as to their perceptions regarding the differences between the use of the MMI and the traditional interviews in the domains of fairness, stress level, and effectiveness in evaluating their non-cognitive traits. These surveys were submitted to a departmental secretary who had no knowledge of their applicant file. Interviewers who performed both traditional interviews and MMI interviews were also surveyed anonymously as to their perceptions regarding the differences between the MMI and traditional interview in terms of fairness, effectiveness in assessing non-cognitive skills, and preference compared to standard interview formats. Paired *t*-tests were used for comparisons between methods with a *p*-value of 0.05 used to determine significance.

The data from the interviews were entered into an Excel database, which was then restructured to facilitate the univariate generalized linear model (GLM). Once positioned, the data were imported into SPSS for analysis and the model GLM was run using the score obtained from the interview as the dependent variable, and the candidate, station, and interviewer as random effects. Random effects models were used to compute estimated variance values, which are needed for calculation of the G-coefficients (12). G-coefficients were computed for each individual station as well as combinations of candidate within the station, station within interviewer, and interviewer within candidate.

The study was approved by the Reading Hospital Institutional Review Board as a quality improvement project; therefore, informed consent was not obtained.

## Results

There were five interview stations in the data (Medical Error, Family Meeting, Last Call, Mentor Meeting, and Overloaded Census), which were fully crossed by both interviewer and candidate. Seventeen interviewers performed a total of 1,185 interviews. There were 237 candidates each of whom participated in all five interview stations.

G-coefficients are reported for each station and ranged from a minimum of 0.9797 for the Last Call station to a high of 0.9848 for the Overloaded Census station (Table 1). G-coefficients for the combinations of candidate within station, station within interviewer, and interviewer within candidate were 0.9615, 0.9814, and 0.9548, respectively.

**Table 1:** G-coefficients for MMI subscales as well as combined reliability

Category	G-coefficient
Medical error	0.9819
Family meeting	0.9832
Last call	0.9797
Mentor meeting	0.9829
OC	0.9848
Candidate within station	0.9615
Station within interviewer	0.9814
Interviewer within candidate	0.9548

Of the 237 applicants who were interviewed, 180 (76%) returned the anonymous survey. Applicants indicated that they agreed with the statements that ‘the MMI was fair’ more strongly than with ‘a traditional interview is fair’ (5.12 vs. 4.07, *p*<0.001) (Table 2). They had higher agreement that ‘the MMI is effective at evaluating non-cognitive skills’ than for ‘the traditional interview is effective evaluating non-cognitive skills’ (5.05 vs. 3.41, *p*<0.001). There was no difference in perceived stressfulness of the MMI compared to the traditional interview (3.06 vs. 3.18, *p*=0.32). The mean agreement with the statement that ‘the MMI process was enjoyable’ was 5.32 on a six-point Likert scale.

**Table 2:** Applicant survey data with selected paired *t*-tests as indicated (*n*=150 for each item)

Survey question	Mean (SD)	*p*
Traditional interview is fair	4.07 (1.26)	
MMI is fair	5.12 (0.79)	<0.001
I enjoyed the MMI	5.32 (0.81)	
Traditional interview is stressful	3.18 (1.34)	
MMI is stressful	3.06 (1.39)	0.32
Traditional interview is effective evaluating non-cognitive skills	3.41 (1.31)	
MMI is effective evaluating non-cognitive skills	5.05 (0.77)	<0.001

All eight interviewers who had experience performing both a traditional interview and the MMI model returned the interviewer survey (100%). Interviewers agreed with the statements regarding fairness of the MMI more strongly than with statements regarding fairness of the traditional interview (5.44 vs. 3.38, *p*=0.01) (Table 3). Interviewers noted higher agreement with statements of the effectiveness of the MMI at evaluating non-cognitive skills than a similar statement regarding the traditional interview (5.44 vs. 3.25, *p*<0.002). Interviewers also had higher agreement with a statement that the MMI was enjoyable than a statement that standard interviews were enjoyable (5.75 vs. 4.25, *p*<0.008). Faculty interviewers were in favor of continuing using the MMI as a recruitment tool in our residency program (average of 5.88 on a six-point Likert scale).

**Table 3:** Interviewer survey data with selected paired *t*-tests as indicated (*n*=8 for each item)

Survey question	Mean (SD)	*p*
Traditional interview is fair assessment tool in screening applicants	3.38 (0.74)	
MMI is fair assessment tool in screening applicants	5.44 (1.05)	*p*=0.01
Traditional interview is enjoyable for me	4.25 (1.04)	
MMI is enjoyable for me	5.75 (0.71)	*p*=0.01
Traditional interview is effective tool in screening applicants	3.25 (0.46)	
MMI is effective tool in screening applicants	5.44 (0.90)	*p*=0.002
I am in favor of continuing to use MMI instead of traditional interviews	5.88 (0.35)	

## Discussion

Our study investigated the reliability and acceptability of a five-station MMI model for internal medicine residency program recruitment. Our generalizability data showed that even with only five stations, the reliability of our process was high enough for high-stakes decisions such as admissions (>0.9 for candidate within station). Prior research has demonstrated similar high reliability values of the MMI model, although using more stations. Eva et al. have found reliability coefficients of 0.73, 0.76, and 0.85 using 8, 9, and 12 stations respectively (12, 15, 19). Roberts et al. described a reliability coefficient of 0.7 on an eight-station MMI study (17). Hofmeister et al. reported a reliability value of 0.67 with the use of 12 stations (21). Our research adds to what is known by demonstrating acceptable reliability for high-stakes decisions (>0.9) using fewer MMI interviews, which may be beneficial to residency programs with fewer available personnel and resources for the interview process.

We also demonstrated that the MMI process was acceptable to a pool of interviewers and interviewees previously exposed to standard interview formats. Interviewees did not find the process more stressful and felt that it was fairer and more effective tool to evaluate their non-cognitive traits. Interviewers echoed these feelings and preferred it to standard interviews. Several studies have confirmed this finding about the MMI experience (13, 14, 18, 22). Hofmeister et al. reported evidence of acceptability of the MMI process in a group of 74 international medical graduates applying to a family medicine residency program and interviewers in Alberta, Canada (14). Dore et al., in a group of 484 Canadian and international medical graduates to three residency programs in Canada, reported that 88% of candidates believed they could accurately portray themselves during the MMI, and 74% of interviewers believed the MMI outperformed the traditional interview (22). Reading Hospital interviewers were highly in favor of continuing the utilization of MMI for resident recruitment. This does not appear to be secondary to a time-saving bias as the total time spent in the interview process using MMI was 90 minutes per interviewer, whereas the previously used traditional interview style required 80 minutes per interviewer.

Our study limitations include the fact that it was conducted in a single institution, and that the number of interviewees was relatively small, so the positive survey findings may be attributable to other aspects of this faculty or applicant pool. Interviewers had undergone training for the MMI during which a case was made regarding the limitations of standard interviews; those interviewers also assisted in developing the cases, making it more likely that they would believe in the process and possibly biasing their answers against standard interviews. Finally, interviewees interested in our program may very well have felt ‘obligated’ to give positive feedback regarding the MMI process, possibly causing a response bias in favor of the MMI.

In conclusion, the MMI process can generate reliable interview results using only five stations at the residency level, and it was found to be acceptable and preferred over standard interview modalities by applicants to one US residency program and its faculty. Whether this five-station MMI process can predict residents who have communication or professionalism problems in residency or in practice as the longer MMIs have (19, 20) and whether our findings can be replicated at larger US residencies are matters for further study. A multi-center study with residency programs of various sizes in both community and university settings is needed to verify our findings.
